# Statins as Modulators of Regulatory T-Cell Biology

**DOI:** 10.1155/2013/167086

**Published:** 2013-10-03

**Authors:** David A. Forero-Peña, Fredy R. S. Gutierrez

**Affiliations:** Grupo de Investigación en Ciencias Biomédicas, Facultad de Medicina, Universidad Antonio Nariño, Bogotá, Colombia

## Abstract

Statins are pharmacological inhibitors of the activity of 3-hydroxy-3-methyl-glutaryl-CoA reductase (HMGCR), an enzyme responsible for the synthesis of cholesterol. Some recent experimental studies have shown that besides their effects on the primary and secondary prevention of cardiovascular diseases, statins may also have beneficial anti-inflammatory effects through diverse mechanisms. On the other hand, the induction and activity of regulatory T cells (Treg) are key processes in the prevention of pathology during chronic inflammatory and autoimmune diseases. Hence, strategies oriented towards the therapeutic expansion of Tregs are gaining special attention among biomedical researchers. The potential effects of statins on the biology of Treg are of particular importance because of their eventual application as *in vivo* inducers of Treg in the treatment of multiple conditions. In this paper we review the experimental evidence pointing out to a potential effect of statins on the role of regulatory T cells in different conditions and discuss its potential clinical significance.

## 1. Introduction

Statins are pharmacological inhibitors of conversion of 3-hydroxy-3-methyl-glutaryl-coenzyme-A (HMGCoA) into L-mevalonate through competitive inhibition at the active site of the HMGCoA reductase. This inhibition results in decreased synthesis of cholesterol and its metabolically active steroidal and no steroidal derivatives [1, 2] and increased expression of LDL receptors in the liver, leading to enhanced clearance of cholesterol from the blood. 

Regulatory T cells (Treg) are a subpopulation of effector T cells devoted to the maintenance of immune tolerance, and hence, their expansion and maintenance are critical in the resolution of inflammation and in preventing sustained tissue damage or autoimmunity. These cells are characterized by the expression of the transcription factor foxp3, which is the master regulator of the immune suppressive activity of Treg. Therefore, the mechanisms that induce foxp3 expression and sustain Treg activity have gained a great interest in recent biomedical research [3, 4].

Some experimental studies have shown that statins may have anti-inflammatory effects that can be observed early after starting the treatment, without affecting blood cholesterol [5, 6]. Indeed, according to a retrospective cohort study including 229,918 adults in Israel, the continued treatment with statins provides a continuing reduction in mortality among patients with and without a known history of coronary heart disease (CHD) [7]. 

Statins significantly reduced the serum level of markers of inflammation (C-reactive protein and serum amyloid A) in patients with atherosclerosis [8]. Plasmatic levels of other intermediate metabolites of cholesterol, including isoprenoids, farnesylpyrophosphates, and geranylgeranylpyrophosphates, are reduced during statins therapy [9]. These effects of statins may be responsible for the benefits observed in experimental studies using animal models of inflammatory and autoimmune diseases [10–12].

Downstream from L-mevalonic acid are the isoprenoids farnesylpyrophosphate (FPP) and geranylgeranylpyrophosphate (GPP), which are involved in posttranscriptional modifications of intracellular proteins, a process that is crucial for intracellular signaling [13, 14]. After inhibition of HMGCoA, the synthesis of isoprenoids is inhibited, and this appears to be a common mechanism that may account for the immune modulatory effects of statins, although other mechanisms may be involved. Isoprenoids participate in various cellular processes, such as the coupling of glycoproteins, proteins binding to Heme groups, and GTP and cell proliferation [15].

Recently, a number of anti-inflammatory effects of statins have been described which have renewed the interest of researchers in the molecular responses modulated by statins. Endothelial dysfunction has been described as a crucial process in the development of cardiovascular diseases. As a consequence, most research attention has been focused on the endothelial effects of statins [16]. Of note, statins are able to inhibit the intracellular signaling mediated by the Rho-kinases, Rac, and Cdc42 that are responsible for the prothrombotic and proinflammatory functions of endothelial cells [17], leading to a reduced immune cell migration, which is a crucial process during the early induction of the immune inflammatory response. Moreover, it was revealed that statins can significantly increase the expression of CCN3 (a member of the CCN family of proteins that participate in the regulation of endothelial dysfunction) and inhibit the expression of vascular cell adhesion molecule-1 (VCAM-1) and the adhesion of monocytes to endothelial cells, via the transcription factor pathway Kruppel-like factor-2 (KLF-2), which results in reduced endothelial inflammation [18].

Nonetheless, the activity of statins in modulating the immune response involves a growing number of mechanisms that have been recognized from *in vivo* and *in vitro* studies in experimental models. Among the mechanisms being proposed to be involved in the immune regulatory properties of statins are the upregulation of endothelial nitric oxide synthase (NOS3), reduced activation of endothelial cells, induction of antioxidative responses, reduced migration of inflammatory cells, reduced antigen presentation due to substantially suppressing the expression of class II MHC in endothelial cells [19], which is mediated via the inducible promoter IV of the class II transactivator (CIITA) [20], reduced expression of proinflammatory cytokines, reduced aggregation of platelets, promotion of 15-epi-lipoxin, an aspirin-triggered eicosanoid derived from arachidonic acid which acts by blocking the signaling pathways involved in the activation of the inflammatory response triggered by lipid mediators [17, 21], and, finally, enhanced migration, differentiation, and suppressive activity of regulatory T cells (Treg) [17, 22]. In this paper we review the potential effect of statins on Treg cell biology, addressing experimental evidence from *in vitro* and *in vivo* studies performed in animal models (summarized in Table 1) and human cells from patients with autoimmune and inflammatory diseases (see Table 2), and discuss the significance of these findings and the controversial use of statins in the treatment of inflammatory or autoimmune diseases and cancer.

## 2. Statins Affect the Microenvironment Required for the Induction and Maintenance of Treg Cells

It is well accepted from the recent scientific literature that IL-6 signaling through the STAT-3 leads to the inhibition of the transcription of foxp3, thus the potential inhibition of IL-6 signaling by statins could lead to indirectly favoring the expression foxp3 and, consequently, an increased percentage of Treg [4]. One of the early effects of IL-6 is the induction of expression of chemoattractants like MCP-1. In fact, statins were able to suppress the chemotaxis of monocytes that is mediated by IL-6/sIL-6R engagement and the expression of MCP-1 expression in cultured human aortic endothelial cells (HAECs). The mechanism behind these antichemotactic properties of statins is by means of inhibiting the phosphorylation of JAK1, JAK2, TYK2, STAT1, and STAT3 and the translocation of STAT3 IL-6/sIL-6R to the nucleus. These effects of statins are mediated by the geranylation of the JAK/STAT molecules, since coincubation of mevalonate and geranylgeranyl pyrophosphate, but not farnesyl pyrophosphate, reversed the inhibitory effects of statins on MCP-1 expression [23]. Hence, statins efficiently block the effects of IL-6 as a consequence of the inhibition of prenylation of STAT3, which is downstream of the signaling triggered by the IL-6 receptor [23]. 

It was reported in PBMCs from patients with chronic idiopathic urticaria (CIU) and from healthy control individuals that simvastatin and lovastatin are strikingly effective in inhibiting the proliferative response of T cells to T-B polyclonal stimuli, *Staphylococcus aureus* enterotoxin A, and the antigen-specific proliferative response. These effects of statins were mainly mediated by the induction of cell cycle arrest in the G0/G1 phase. Interestingly, when cultured in the presence of PHA plus simvastatin, PBMC from both patients and healthy individuals exhibited significantly reduced production of IFN-*γ*, IL-10, and IL-17A, indicating a broad effect of statins in the induction of effector T-cell responses. Simvastatin and lovastatin were also effective in reducing the levels of TNF-*α*, IL-6, and MIP-1*α* produced by human PBMC after being cultured in the presence of LPS, although the same effect was not observed in LPS-stimulated monocytes purified from the same individuals. Conversely to what will be discussed below, in this study, statins did not have a significant effect in Th17 responses, since the levels of mRNA for RORC-*γ*t were unaffected in both patients and controls, but the statins significantly increased the levels of mRNA transcripts for SOCS-3, however, only in healthy controls. Conversely, the levels of mRNA for indole-amine-2, 3-dioxygenase (IDO) were slightly reduced in PBMC from CIU patients [24].

Statins may be able to modulate antigen presenting cells (APC) to favor the development of Treg. In fact, in experimental autoimmune myasthenia gravis (EAMG), Li et al. demonstrated that DC can be turned into tolerogenic DC (tDC) after the treatment *in vitro* with atorvastatin. Moreover, the administration of these statin-induced tDC in mice with EAMG results in clinical improvement, with increased numbers of Treg cells [25].

## 3. Statins Directly Enhance the Induction of Treg Cells

In addition to the vast body of experimental evidence that has been collected in the last years indicating several anti-inflammatory roles of statins, these medications may also directly potentiate the induction of Treg. In fact, this mechanism may be behind the cardiovascular protective effects of statins, as it has been demonstrated that foxp3^+^ Treg cells inhibit the development of atherosclerosis by modulating the metabolism of lipoproteins [26]. When Tregs were depleted in transgenic mice, they showed increased levels of plasma cholesterol and increased atherosclerosis [27]. Atorvastatin and simvastatin induce increased numbers of Tregs in atherosclerotic plaques from patients with acute coronary syndrome [28, 29], which correlates with a better prognosis in patients with myocardial infarction with elevated ST-segment before primary percutaneous coronary intervention [29].

Of note, in a model of ischemia-reperfusion myocardial injury in mice, the prior intravenous administration of a high dose of rosuvastatin significantly increased the expression of foxp3 in the heart and spleen and enhanced the accumulation of Tregs in heart, leading to significantly reduced serum levels of cardiac troponin I and reduced infarct sizes when compared to those of control mice, an effect that was completely reversible by mevalonate, suggesting that rosuvastatin not only can induce recruitment of Tregs in ischemic areas but also is capable of increasing the differentiation of Tregs in the myocardium [30]. Taken together, these studies are in support of the concept that part of the therapeutic benefit of statins in cardiovascular diseases is related to the effect of these medications on the function of Treg cells.

The direct induction of Treg by statins was also shown *in vitro* using cultures of mice [31] and human [32] peripheral blood mononuclear cells (PBMCs). Atorvastatin, but not mevastatin nor pravastatin, significantly increased the number of CD4^+^CD25^high^ cells and CD4^+^CD25^+^Foxp3^+^ cells which were correlated with an increased regulatory activity [32]. In addition, individuals that received simvastatin and pravastatin for hyperlipidemia exhibited increased numbers of circulating Treg, suggesting that statins may also act *in vivo* favoring the differentiation of T cells into Treg [32, 33]. Although this study failed to demonstrate a direct effect of statins in inducing increased numbers of Treg in mice [32], another study using atorvastatin in patients with acute myocardial infarction with ST-segment elevation before performing a primary percutaneous coronary intervention demonstrated that the statin significantly increased the numbers of CD4^+^CD25^+^ Tregs and the expression of foxp3 in PBMC from the patients, which was associated with an increased production of TGF-*β* and a decreased INF-gamma [29]. Similar results were also found in PBMC from patients with acute coronary syndrome, in which adding simvastatin to the culture led to an increased proportion (and suppressive function) of Tregs [28].

Statins are also capable of potentiat Treg differentiation and maintenance by influencing transcription factors that are central to Treg development. Indeed, a different group of researchers found that simvastatin is able to control the methylation of the promoter region of foxp3 and also this statin inhibits the induction of Smad6 and Smad7, therefore releasing and potentiating TGF-*β* signaling. As a consequence, simvastatin facilitated the induction of foxp3 expression in T cells obtained from TCR transgenic mice on a RAG−/− background, after being cultured in the presence of anti-CD3/D28 and rIL-2. This is mediated through a synergistic effect with TGF-*β* [34]. These effects were also mediated by the inhibition of geranyltransferase [34].

## 4. Statins Are Able to Influence the Migration of Treg Cells

The anti-inflammatory effects of statins can involve a direct inhibition of leukocyte migration. Lymphocyte Function-Associated Antigen-1 (LFA-1) is an integrin that is widely expressed in hematopoietic cells, and its main ligand is Intracellular Adhesion Molecule-1 (ICAM-1), which is induced on the surface of endothelial cells in response to gamma-interferon (IFN-*γ*). During early steps of inflammation, the engagement of LFA-1 by ICAM-1 is critical. In a report, lovastatin, simvastatin, and mevastatin, but not pravastatin, were able to bind to an allosteric site of LFA-1, thereby preventing it from interacting with ICAM-1, leading to a disruption of the interaction between leukocytes and the endothelium, with a consequent impact on the migration of neutrophils in a model of peritonitis in mice [35]. 

Furthermore, statins can also directly promote the migration of Treg to inflamed tissues. It was shown that lovastatin increases the amounts of Treg into skin lesions in a model of DTH induced by *Candida albicans*, by increasing the expression of CCL1,a chemokine that participates in the chemotaxis of Treg [36].

## 5. Statins Induce Increased Homing of Treg Cells to Inflamed Tissues

In addition to enhancing the induction of Treg, statins could also potentiate the selective migration of these suppressor cells to the site of inflammation. Indeed, in a study using a mouse model of DTH, it was found that lovastatin significantly reduces the mRNA levels of proinflammatory cytokines (IFN-*γ* IL-12, IL-1*α*, IL-1*β*, and TNF-*α*) while increasing mRNA for IL-4, IL10, and TGF-*β*. In line with this, lovastatin also modulated a number of chemokines and chemokine receptors and induced increased migration of Tregs to the inflamed area, which was dampened in CCL1−/− mice. Of note, CCL1 is a ligand for the chemokine receptor CCR8, and FoxP3^+^ cells expressing CCR8 have been found to be present in inflamed tissues and draining lymph nodes in mice [36]. These findings suggest that statins may induce a selective migration of Treg to the site of inflammation, in response to the chemoattractant CCL1.

These effects of statins in favoring the accumulation of Treg in inflamed tissues were also observed in a model of atherosclerosis in apo lipoprotein E-deficient (ApoE−/−) mice, and simvastatin increased the expression of Foxp3, IL-10, and TGF-*β* in both the atherosclerotic plaque and in circulating cells. In addition, simvastatin induced increased presence of IL-4, along with decreased IL1-*β*, IFN-*γ*, and IL-17 in the plaque, as measured by qPCR and quantitative immunohistochemistry [28].

## 6. Statins Inhibit the Induction of Th1 Cells

One of the possible mechanisms by which statins could influence the activity of Treg is through the inhibition of the Th1 immune response. In line with this, statins may modulate at different levels the induction of Th1 cells. One of them involves the modulation of the expression of CD40, which is a member of the TNFR superfamily with specificity for CD40 ligand. It is found on mature B lymphocytes and some epithelial cells, as well as in lymphoid dendritic cells. It induces the expression of IL-12 which in turn promotes the differentiation and clonal expansion of helper T cells into Th1 cells [37]. Atorvastatin reduces the expression of CD40 and IFN-*γ* induced production of TNF-*α* on human endothelial cells and monocytes [38]. Interestingly, this effect was reversed by the administration of exogenous mevalonic acid, suggesting the existence of another mechanism of action, independent of HMG CoA reductase inhibition [38]. This might be related to the report of a new statin allosteric site of LFA-1 for statins, as demonstrated in a study on T-cell proliferation assays [35].

The treatment with statins has been demonstrated to improve the survival in septic mice. It has been proposed that sepsis induced increased activity of farnesyltransferase, and consequently higher levels of farnesylated proteins were found in association with an increase in the numbers of Tregs, suppressed secretion of IFN-*γ*, and an attenuated T-cell proliferation. As statins act as inhibitors of farnesyltransferase [39], it has been proposed as a potential drug in the immunomodulation of sepsis [40, 41]. In fact, in a study using the experimental model of sepsis induced by cecal ligation and puncture (CLP), the treatment of septic mice with the farnesyltransferase inhibitor FTI-277 led to reduced mortality and improved bacterial clearance. Interestingly and contrary to what is observed in healthy mouse, the FTI-227 produced a reduction in the number of circulating Tregs in septicemic mice [39]. In addition, simvastatin attenuated CD4 T-cell apoptosis induced by sepsis and promotes proliferation of these cells and the production of cytokines, and contrary to the effect of the treatment in healthy mice, simvastatin reduces the expansion of regulatory T cells in septic littermates, which correlated with a reduced bacteremia in septic animals, suggesting that this attenuation in Tregs, mediated by simvastatin, contributes to the beneficial action of statins in patients with severe infections [42]. These results indicate that the inhibition of farnesyltransferase by statins exerts different effects on the balance of Th1 and Th2 responses and constitutes a central mechanism of modulation of T-cell immune dysfunction during sepsis. 

HMGCoA reductase inhibitors interfere with the differentiation of lymphocytes in the Th1 and Th17 phenotypes and produce a marked increase in the differentiation of FoxP3^+^CD4^+^ T cells *in vitro* and *in vivo*. Similarly, using a geranylgeranyltransferase inhibitor (GGTI-289) and farnesyltransferase inhibitor (FTI-227), it was found that GGTI-289 but not FTI-227 mimics the effect of simvastatin, suggesting that inhibition of geranylgeranylation protein is responsible for the reduced differentiation of Th17 cells and the increased differentiation of CD4^+^FoxP3^+^. In addition, it was also demonstrated that GGTI-289 induces the expression of SOCS3 (suppressor of cytokine signaling 3, a protein involved in the negative regulation of signaling cytokines through the JAK/STAT), thus inhibiting the phosphorylation of STAT3 in CD4^+^ T cells induced by IL-6 signaling. This gives us strong evidence that the balance between the differentiation of Th17 and Foxp3^+^ Tregs is mediated by the protein geranylgeranylation through increased expression of SOCS3 [43].

## 7. Statins Inhibit the Induction of Th17 Cells

The opposing functions of Th17 immune cells and Treg have led many researchers to propose that unbalanced Treg/Th17 responses may be involved in the pathogenesis of chronic inflammatory diseases. Interestingly, statins are also able to inhibit the development of Th17 cells, potentiating their effect on Treg functions.

It was found in spleen cells from BALB/c mice cultured under different polarizing conditions that simvastatin inhibits the differentiation of Th17 cells and enhances the differentiation of foxp3^+^CD4^+^ T cells [43]. Protein geranylgeranylation appears to be the mechanism by which the balance between the differentiation of Th17 cells and Treg cells is regulated, since geranylgeranyltransferase inhibitor GGTI-298 but not the inhibitor of farnesyltransferase FTI-277 mimicked the effects of simvastatin. These GGTI-induced Tregs were effective in suppressing the immune response in an experimental model of induced colitis in SCID mice [43].

Likewise, atorvastatin markedly improves the manifestations of experimental autoimmune (EAN), an acute inflammatory disease of the peripheral nervous system commonly used as a model for Guillain-Barre. A decreased infiltration of inflammatory cells into the peripheral nervous system and a decreased percentage and number of INF-*γ* and IL-17 producing lymphocytes in the sciatic nerve were found after treatment of mice with simvastatin [44]. Additionally it was shown that atorvastatin decreases the expression of costimulatory molecules for T-cell activation such as CD80 and increases the number of Tregs in mononuclear cells lymph nodes [44]. Therefore, these data suggest that atorvastatin can act as an inhibitor of inflammatory Th1 and Th17 responses during EAN, decreasing the expression of costimulatory molecules as well as increasing the number of Tregs. 

In human CD14^+^ cells separated from PBMC of patients with relapsing remitting multiple sclerosis as well as of control individuals, cultured in the presence of simvastatin, it was shown that the statin induces the expression of SOCS3 and SOCS7, consecutively inhibiting the phosphorylation of the transcription factors STAT3 and STAT1 that are involved in the regulation of IL-6 and IL-23. Furthermore, simvastatin was also able to induce the secretion of INF-*γ*, IL-4, and IL-27 and suppressed the expression of the transcription factor RORc and the secretion of IL-17 by CD4^+^ T cells that were isolated from the same individuals [45]. 

Using the same approach on cultured naive CD4^+^ cells derived from patients with relapsing remitting multiple sclerosis, other authors found that simvastatin inhibits Th17 cell differentiation and the secretion of the cytokines IL-17A, IL-17F, IL-21, and IL-22 by *in vitro* differentiated naive CD4^+^ T cells from patients [46].

The development of human Th17 cell differentiation largely depends on the action of IFN regulatory factor 4 (IRF4), which in turn is phosphorylated by the RhoA-associated kinase (ROCK) 2, triggering the binding of IRF4 to the IL-17A and IL-21 promoters and thus inducing their activation. The authors used IRF4 gene knockdown and overexpression experiments to demonstrate that this inhibition of Th17 cell differentiation was mediated by the inhibition of IRF4 expression [46]. This effect mimicked that observed with the Rho/ROCK inhibitor Y-27632, then suggesting that inhibition of IRF4 is a critical process in the regulation of Th17 by simvastatin.

In addition to a reduced number of Tregs, the impairment of their suppressive activities may play a role in the pathogenesis of some autoimmune diseases. That is the case in patients with rheumatoid arthritis, in whom the PBMC exhibited a normal number of Tregs and foxp3 expression but reduced suppressive activities of these cells when compared to healthy controls [47]. Moreover, a significant increase in the numbers of circulating Tregs was observed in patients treated with atorvastatin for 12 weeks. These findings correlated with an increase in the suppressive function of Tregs compared to individuals that did not receive the statin. As expected, these results were accompanied by a significant reduction of disease activity (measured by Disease Activity Score 28, erythrocyte sedimentation rate, and high sensitive C-reactive protein) [47].

These findings were confirmed by experiments in which atorvastatin was added to cultured CD4^+^CD25^−^ cells isolated from PBMC from patients suffering from rheumatoid arthritis. A significant, dose-dependent increase in the percentage of CD4^+^CD25^+^Foxp3^+^ cells was observed in the atorvastatin treated cultures that was reversed by adding L-mevalonate to the cells. Further experiments demonstrated that the effects of atorvastatin in Treg were mediated by the induction of reduced phosphorylation of Akt, mTOR, and ERK. These kinases are known substrates of the small GTPases Ras and Rho-GTPases and participate in the signaling pathway that regulates the expression of Foxp3 by activated and naïve T cells [4]. Thus, these data evidenced a role of these kinases in the mechanism of action of statins on Treg induction.

In asthma, it was found that cultured CD4 T cells and monocyte-derived dendritic cells (mDC) from asthmatic patients, when incubated in the combination of corticosteroids (fluticasone propionate (FP)) and a statin (simvastatin), exhibited a greater differentiation into Tregs *in vitro*, along with a reciprocal reduced numbers of Th17 cells when compared with the treatment with simvastatin or FP alone. The mechanisms related to this additive effect of the combination of medications were an enhanced production of IL-10 and IDO and reduced IL-23 [48].

## 8. Statins as Potential Adjuvants in the Treatment of Chronic Inflammatory and Autoimmune Diseases

Aside from the above described effects on transcription factors influencing Treg development and maintenance, statins are also able to reduce the inflammatory response by decreasing the production of proinflammatory cytokines INF-*γ*, TNF-*α*, IL-23, and IL-6, as well as monocyte chemotactic protein (MCP-1) as was shown in leukocytes from the ileum of mice treated with simvastatin and infected with *Toxoplasma gondii*. As expected these changes in the production of cytokines were in association with increased numbers of Tregs and increased concentrations of IL-10 in the ileum, in the mesenteric lymph nodes and in the spleen [49].

The beneficial effects of statin therapy in renal inflammatory disease may also relay in the enhancement of IL-production 10 and not in a direct effect on the numbers or activity of Tregs. In a study using experimental autoimmune glomerulonephritis, atorvastatin had no direct effect on the suppressive activity or expansion of Treg, but it significantly increased the secretion of IL-10 by Treg cells, reduced the numbers of kidney-infiltrating macrophages, CD4 T cells, and Th17 cells, and reduced the production of the cytokines IL-17 and IFN-*γ*, leading to an improved renal function, decreased albuminuria, and ameliorated histological changes in antiglomerular basement membrane (GBM) glomerulonephritis (GN) [50].

Statins may also act as adjuvants in the treatment of experimental inflammatory conditions. In fact, two independent studies on a model of experimental autoimmune encephalitis (EAE) evidenced that atorvastatin or lovastatin was able to slow or suppress the evolution of the disease in a dose-dependent manner [51, 52], when used in combination with two immune modulator drugs in clinical use (rolipram and glatiramer acetate). 

It was also found that the combination results in a decreased secretion of proinflammatory cytokines (IL-12, INF-*γ*, and TNF-*α*) and NO and increased secretion of Th2 cytokines by monocytes (IL-4, IL-10, and TGF-*β*) [51, 54], while suppressing the Th1 response [54–56]. These findings were associated with a significant enhancement of the expression of IDO (which is known to suppress EAE development through suppression of T-cells response and induction of immune tolerance) and a reduced Th17 effector response [52]. 

In accordance, significantly higher levels of expression of TGF-*β*, CD25, and foxp3 were found in the spinal cord of rats treated with lovastatin when compared with animals receiving vehicle. More importantly, this change was strikingly enhanced when lovastatin was combined with rolipram. Meanwhile, rolipram alone was not able to induce substantial changes in the expression of these mediators [52]. These reports strongly indicate that statins complement and synergize with rolipram and glatiramer acetate to reverse the clinical progression of EAE [51, 52], and they suggest that a combination therapy of statins with existing therapeutics in the treatment of multiple sclerosis may have better clinical outcomes than the monotherapy. 

## 9. Potential Risks of Treg Induction by Statins

The effects of statins as modulators of the immune response may turn these medications into detrimental factors influencing the appearance of neurodegenerative disorders, such as amyotrophic lateral sclerosis, and a myriad of infections, including HIV, hepatitis B virus, hepatitis C virus, and varicella zoster virus, among other diseases. Moreover, since Tregs have been related to cancer development and progression by suppressing tumor-specific effector T-cell responses, and statins have systematically been demonstrated to induce Treg, some concerns have been raised about the potential risk of statins usage mainly in the ageing people that are supposedly more prone to develop cancer of different tissues because of the process of immune senescence [57, 58]. These points were addressed to some extent in a report on cultures of tumor cell lines (3LL, A549, and NCI-H292), where simvastatin was able to reduce the number of tumor cells and increase the production of IL-10 and TGF-*β* in a dose-dependent manner, associated with increased expression of IDO and foxp3 and therefore accompanied by increased expansion of Tregs [53].

Further analyses showed that the inhibition of tumor cell growth was due to retention in the G1 phase of the cell cycle by inhibition of the expression of Cyclin D1 (a molecule that regulates progression through this stage of the cell cycle). IDO is an enzyme involved in the metabolism of tryptophan, and its increased activity generates deprivation of this amino acid, which exerts inhibitory effects on the proliferation of T lymphocytes. However, in this study the authors did not find significant differences in tumor growth *in vivo* between untreated and simvastatin-treated mice after the mice were injected with murine cancer cells (3LL) [53]. 

Nonetheless, a more recent study evaluated the mortality of patients from the entire Danish population who were diagnosed with cancer between 1995 and 2007 and made a followup to the patients until December 31, 2009. It was shown that statins significantly reduced the mortality related to cancer when compared with those individuals who had never used statins, and this protection was observed in 13 different types of cancer [59]. Thus, no definitive evidence exists in support of a harmful role of statins therapy in the context of cancer, but further experimental and clinical studies are required to shed light onto these issues. 

In addition, several studies have raised the concern about the fact that statins may also favor the development of diabetes in chronic users. It was shown that statins may increase the hazard ratio for newly diagnosed diabetes among 13–25%. This may be in association with the fact that statins may induce decreased Ca2^+^-dependent insulin secretion and may also interfere with isoprenylation of guanosine triphosphate (GTP) binding proteins in the *β* cells of the pancreas [60]. It has not been, however, established if the mechanism of induction of diabetes during therapy with statins directly involves the immune response.

## 10. Perspectives and Concluding Remarks

Statins have been shown to potentiate the suppression of the immune response mediated by Treg cells, which generally translates in reduced inflammatory response. This could be a therapeutic goal in some inflammatory and autoimmune diseases. However, this effect may not be of benefit in tumor diseases because of the potential risk of tumor progression. 

In the medical setting, as the prescription of statins in patients receiving other therapeutic immune modulators could potentiate the effects of statins in Treg cells, it is necessary for the clinician to know the potential immune modulatory effects when combining these medications, since this effect may increase the risk of reactivation of latent infectious diseases and may also promote tumor progression.

Considering the wide use of these medications and its potentially significant benefits in the therapy of inflammatory and autoimmune diseases, further research is required with a particular emphasis on the molecular targets of statins involved in the regulation of the immune response.

Another field that may be of relevance as an effect of statins is in the modulation of oxidative and nitrosative stress. In line with this, there is evidence of increased oxidatively modified lipoprotein (oxLDLs) in patients with chronic hemodialysis, with a consequent increase of oxidative stress; additionally related oxLDL with a decreased expression of foxp3 in CD4^+^/CD25^+^, which promotes a reduction in the surprising ability of Tregs and consequently accelerates the atherosclerotic process in these patients. Therefore statins may be useful to induce the expression of Foxp3 and consequently could reduce the cardiovascular risk in patients with chronic renal disease [26].

In the context of Graft-versus-host disease (GVHD), it is suggested that the thymus-derived Tregs (nTregs) are stable in suppressing the acute inflammatory environment GvHD. Therefore the therapeutic goal would be based on the expansion of alloantigen-specific nTregs. Thus, as statins are able to induce enriched differentiation of Treg, these medications may be of use to this purpose [61].

An increasing number of epidemiological and genetic studies have proven strong lines of evidence for a close connection between the pathophysiology atherosclerotic cardiovascular disease (ACE) and chronic inflammatory diseases [62]. Thus, a fertile field exists for the expansion of Treg cells with the intention of an adoptive transfer as a valid therapy for various inflammatory diseases. Besides the use of monoclonal antibodies, recombinant cytokines led to increasing the Treg cell population or their activity *in vitro*; statins are a family of promising drugs that could also facilitate this expansion specifically *in vivo*.

In conclusion, statins are able to modulate some key mechanisms that control the immune response, as shown in *in vitro* and *in vivo* studies using models of autoimmune diseases and in studies on human cells. However, the potential applications of statins on these conditions are still controversial and their successes are largely dependent on the careful conduction of clinical and preclinical studies on this matter.

## Figures and Tables

**Table 1:** Lines of evidence on the modulation of regulatory T cells by statins in mice.

Statin	Dose	Experimental model	Possible mechanism involving Treg	Ref.
Atorvastatin	1–10 mg/kg	Experimental autoimmune neuritis	↑ Treg number ↓ Inflammatory cells ↓ INF-*γ* ^+^ and IL-17^+^ cells ↓ CD80 expression	[44]

Atorvastatin	10 mg/kg	Experimental autoimmune glomerulonephritis	↑ IL-10, ↓ IL-17, IFN-g ↓ TNF-*γ*↓ IL-21 *↕* Treg	[50]

Atorvastatin	*In vitro*: 10 mM	Experimental autoimmune myasthenia gravis	↑ Foxp3 expression, ↑ Treg number ↓ Lymphocyte proliferation ↓ Th1/Th17, ↑ Th2	[25]

Lovastatin	10 mg/kg i.p	DTH (*C. albicans*)	↑ Migration of Tregs to the foot-pad ↑ CCl1 and IL-10	[36]

Rosuvastatin	5 mg/kg i.v.	Ischemia-reperfusion injury	↑ Foxp3 expression	[30]

Simvastatin	10, 50 mg/kg	Tumor growth of 3LL cell line	↑ Fox-p3, IDO, IL-10, and TGF-*β*	[53]

Simvastatin	*In vitro*: 0.5–2 *µ*M	Treg differentiation	↑ Differentiation of Treg ↑ Foxp3 expression ↓ SMAD6 and SMAD7 ↓ Methylation of foxp3 promoter	[34]

Simvastatin	*In vitro*: 2 *µ*M	Treg/Th17 differentiation.	↑ Differentiation of CD4 into Treg ↓ Differentiation of Th17 ↑ SOCS3	[43]

Simvastatin	20 mg/kg	*Toxoplasma gondii *small intestine inflammation	↓ INF-*γ*, TNF-*α*, IL-23, and IL-6 ↑ Foxp3^+^ cells ↑ IL-10 ↓ Neutrophils, and MCP-1	[49]

Simvastatin	50/mg/kg	ApoE−/− mice model of atherosclerosis	↑ Treg cells number ↑ Foxp3 expression ↓ IL-1*β*, IL-17, and INF-*γ* ↑ TGF-B, IL-10, and IL4	[28]

**Table 2:** Evidences on the modulation of Treg cells by statins in humans.

Statin	Dose	Subjects and approach	Possible mechanism involving Treg	Ref.
Atorvastatin	20 mg/day for 1 month	Two groups: 25 stable renal transplant recipients with LDL > 100 and 25 hypercholesterolemic patients with LDL level above target values. Measures were done before and after treatment	↓ Lymphocyte proliferation ↑ ATP in CD4^+^ T cells *↔* Cytokines	[33]

Atorvastatin	*In vitro*: 10 mM	Human endothelial cell culture	↓ Expression of CD40	[38]

Atorvastatin	*In vitro*: 5–10 mM	PBMC culture	↑ Treg cells number ↑ Suppressive function of Tregs	[32]

Atorvastatin	*In vitro*: 10 mM *In vivo*: 20 mg/d	PBMC from patients with RA	↑ Treg cells number ↑ Treg cells suppressive function ↓ Phosphorylation of Akt, mTOR, and ERK	[47]

Atorvastatin	80 mg	Blood samples obtained from the ICA and a peripheral vein, STEMI patients	↑ Number of CD4^+^ CD25^+^ Tregs ↓ INF-*γ* ↑ Expression of Foxp3 ↑ TGF-*β*	[29]

Simvastatin/lovastatin	0.5–25 mM	PBMC from patients with CIU	↓ T-cell proliferation ↓ IL-10 and Il17 *↔* Neither Socs3 nor RORc	[24]

Simvastatin	*In vitro:* 10 nM	PBMCs from MS patients and healthy controls	↓ IL-6 and IL-23 ↑ INF-*γ*, IL-4, and Il-27 ↓ RORC and IL17 gene expression ↑ SOCS3	[45]

Simvastatin	*In vitro*: 10 mM	PBMC from SCA patients	↑ Treg cells number ↑ Treg cells suppressive activity	[28]

Simvastatin	*In vitro*: 10 mM	Naive CD4^+^ T cells derived from MS patients	↓ Th17 differentiation ↓ IL-17A, IL-17F, and IL-21 ↓ IRF4 phosphorylation ↓ Rho/ROCK kinase activity	[46]

Simvastatin	*In vitro*: 10 *µ*M	mDC from asthmatic patients	↓ Th17 cells, ↓ IL-6, ↓ IL-23 ↑ Treg cells ↑ IDO and ↑ IL-10	[48]
